# Olfactory distortions in the general population

**DOI:** 10.1038/s41598-022-13201-5

**Published:** 2022-06-13

**Authors:** Jonas K. Olofsson, Fredrik Ekesten, Steven Nordin

**Affiliations:** 1 Department of Psychology, Stockholm University, Stockholm, Sweden; 2 Department of Psychology, Umeå University, Umeå, Sweden

**Keywords:** Neuroscience, Psychology

## Abstract

Parosmia, distorted smell sensations, is a common consequence of respiratory virus infections. The phenomenon is not well understood in terms of its impact and long-term outcomes. We examined self-reported experiences of parosmia in a population-based sample from the Betula study that was conducted in Umeå in northern Sweden (baseline data collected in 1998–2000). We used a baseline sample of 2168 individuals aged 35–90 years and with no cognitive impairment at baseline. We investigated the prevalence of parosmia experiences and, using regression analyses, its relationship to other olfactory and cognitive variables and quality of life. Benefitting from the longitudinal study design, we also assessed the persistence of parosmia over 5 and 10 years prospectively. Parosmia experiences were prevalent in 4.8% of the population and it often co-occurred with phantosmia (“olfactory hallucinations”), but was not associated with lower self-rated overall quality of life or poor performance on olfactory or cognitive tests. For some individuals, parosmia was retained 5 years (17.0%) or even 10 years later (10.3%). Thus, parosmia experiences are commonly reported in the population, and can be persistent for some individuals, but might be mostly benign in nature. Our work complements research on clinical-level parosmia, which is typically more severe, and recent parosmia reports during the COVID-19 pandemic, where long-term outcomes are still unknown.

## Introduction

Parosmia is an olfactory disorder (OD) where odor perception is distorted; a variety of odor stimuli may trigger unpleasant sensations (i.e. smelling freshly brewed coffee, but experiencing a rotten smell). Parosmia is, together with phantosmia (experiencing an odor without the presence of a triggering source), referred to as qualitative OD^1–3^. Qualitative OD are less well understood than quantitative OD, such as hyposmia—reduced olfactory ability—and anosmia—complete loss of smell^4–6^. Olfactory disorders are common in the general population, especially in the older age-groups, but are often not diagnosed^7^. The total prevalence of OD in the general population was estimated to 28.8% with objective measurements, and about 9.5% with subjective assessments^7^. A Swedish population-based study yielded an estimated parosmia prevalence of 4.0% in the adult population^2^. However, a German study estimated the prevalence of parosmia to be only 1–2%^8^. The variability may be due to differences in how parosmia is assessed.

The olfactory experiences in parosmia are unpleasant; rotten, burnt and sewage are examples of sensations reported by parosmics, and tobacco, coffee, perfume and citrus fruits are some of the most common triggers^9^. The COVID-19 pandemic has led to an increased awareness of, and interest in parosmia. Although patients with parosmia tend to describe their olfactory experiences as sewage or similar, the actual sensation may be novel, and it is triggered by specific molecules^10,11^. Patients resort to illustrating their parosmic sensation with names of odors that are common and unpleasant, to communicate the level of disgust experienced^10^. Olfactory deficits are more common in men than in women^7^. Phantosmia on the other hand may be more common in women^12^. Parosmia is thought to similarly affect men and women^2,5,13^. Some studies found no association between parosmia and age^5,13^, whereas others report higher prevalence in young adults^2^.

Parosmia often co-occurs with a quantitative OD and between 10 and 60% of the OD patient-group suffer from parosmia^4–6,9,13^. Hyposmia is commonly observed among parosmics^4,6,9^. The most common etiologies for parosmia are upper respiratory tract infection (URTI) head trauma, sinonasal disease, neurological deficits, exposure to toxic chemicals, psychiatric connections and nasal operations^5,6,14,15^. COVID-19 is a type of URTI^16^. In a recent comparison between COVID-19-positive patients and patients with a different type of URTI, results showed that both parosmia and hyposmia were more frequent in the COVID-19-positive group^16^. Hence, parosmia and quantitative ODs appear strongly associated, and the COVID-19 pandemic has emphasized the need for understanding their long-term impact. In the present study, we analyze reports of parosmic experiences, using long-term data that were collected before the COVID-19 pandemic, but that may give insights into the duration and impact of parosmia.

Research conducted before the COVID-19 pandemic indicated that 57.1% of parosmia cases develop within a few months following the onset of a quantitative OD^9^. The longitudinal prognosis of parosmia is uncertain, since there is little known about how persistent parosmia is over time. There are records of patients having parosmia for between three months to 22 years, though these are based on retrospective self-evaluations^9^. Parosmia is associated with a lower volume of olfactory cortical regions, as well as behavioral deficits in odor discrimination and odor memory^14^. This morphological difference might help explain the distorted olfactory qualities. In a study examining brain activation, patients with parosmia and hyposmia had a higher activation in areas associated with attention directing (in relation to smells), the processing of odor valence, and the perception of contempt and disgust^14^. Generally, unpleasant odors result in a stronger brain activation response than pleasant odors, possibly due to their higher biological significance^15^. Parosmia may thus either cause, or be a consequence of, changes in cortical pathways. It is critical to understand the persistence of parosmia, since the condition could become chronic for some individuals.

Olfactory disorders have been associated with depression and a reduced quality of life (QoL). Patients with depression often perform worse than healthy controls on olfactory tests^17^. Patients with more severe depression are more likely to report parosmia and/or phantosmia^13^. Between one fourth to one third of individuals with OD exhibited symptoms of depression, whilst about a third experienced a lowered QoL^3^. Furthermore, about one third of individuals with qualitative ODs reported high depression scores on Beck's Depression Inventory (BDI)^3^. Severe self-rated qualitative OD is associated with more severe self-rated depression^13^. About half of parosmic patients reported their quality of life as being severely affected due to their OD^9^. Patients with parosmia and an olfactory deficit (hyposmia/anosmia) have difficulty coping with their olfactory dysfunction, compared to patients with only an olfactory deficit^4^. Mild depression was significantly more frequent in the parosmic group, compared to a control group with only quantitative OD^4^. Patients may adapt to their OD over time, resulting in reduced depressive symptoms at follow-up^3^. Thus, patients with ODs, and especially parosmia, more often experience a lowered quality of life due to their disorder, and are at higher risk of having depression. It is unclear to what extent an OD results in more depressive symptoms or if depression might also be affecting olfactory recovery. While olfactory loss may result in reduced food consumption, but also compensatory overeating^3^, parosmia has negative effects on appetite, mood, or interpersonal relationships^18^. Food flavor is distorted in parosmia, and appetite is diminished^9,18^.

There is a lack of large-scale, prospective studies on parosmia in the general population. Given the current circumstances of the COVID-19-pandemic, it is critical to estimate the persistence and impact of parosmia. We used population-based data from the longitudinal Betula study to investigate the prevalence of parosmia experiences in a large survey sample of adults, and its development over 5 and 10 years. Additionally, we investigated associations between experiences of parosmia and demographic (sex, age, education), olfactory (self-rated ability to perceive weak odors, phantosmia, current disease in nose, performance on the Scandinavian Odor Identification Test, SOIT), cognitive (vocabulary and Mini-Mental State Examination, MMSE) and QoL variables. We defined parosmia based on how participant responded to designated survey questions, not based on a clinical assessment.

The study was conducted with 5 aims. First, we examined the prevalence of parosmia in a population-based sample, with the hypothesis that the prevalence will be around 4%, mimicking the results from a previous study^2^. Second, we investigated the relationship between parosmia and sex, age and education; we hypothesized that these variables would not be associated with parosmia^2,5,13^. Third, we investigated the relationship between parosmia and other olfactory variables, hypothesizing that parosmics will rate themselves having a worse olfactory ability, perform worse on an odor identification test, and report a higher frequency of phantosmia, than non-parosmics^4–6,9,12^. Fourth, we compared QoL scores between the parosmic and non-parosmic population. We hypothesized that those with parosmia will provide lower ratings, since previous studies have shown a strong association between olfactory disorders, including parosmia, and QoL as well as depression and reduced appetite^4,9^. Lastly, we explored how often parosmia persisted over 5 and 10 years and if there were any clear predictors of parosmia longevity.

## Materials and methods

### The Betula study

The present data was collected as part of the Betula study, a prospective population-based study of aging, cognition, and health^19–21^. In Betula, extensive psychological testing and health assessments are conducted every 5 years. The first test wave was conducted in 1988–1990, and as of 2021 the latest wave was implemented in 2017. The Betula study includes assessments on a large number of variables (genetic, lifestyle, health, brain and cognitive) through interviews, tests and questionnaires, which all were administered by trained professionals. The baseline data used in the present study were collected during the third wave of testing which occurred in 1998–2000; this is when olfactory assessments were included in the testing battery. Additional data were obtained from the fourth (2003–2005; 5–year follow-up) and fifth wave (2008–2010; ten-year follow-up) of testing. The data were obtained from age-stratified independent random samples from the population of Umeå, a city with about 110,000 inhabitants, located in northern Sweden. Sample 1 were recruited at the first test wave (1988–1990) of the Betula study and have been part of every test wave since. Sample 2 and Sample 3 were recruited at the second test wave (1993–1995). Sample 2 was designed to mimic sample 1’s age at the first test wave, and was also part of test wave three. Sample 3 mimicked sample 1’s age at the current wave and was also part of all subsequent test waves. Sample 4 was only included at test wave 3, and was selected to mimic sample 1’s age at wave 1. Accordingly, sample 1 were tested on two previous occasions, sample 2 and 3 had experience from one previous test wave, and sample 4 had no previous testing experience. The purpose of recruiting multiple samples was to accurately model cohort-effects on aging and cognition, but here we aggregate data across samples. Participants took part in two in-person sessions per testing wave. Cognitive variables were assessed in an experimental testing session and led by a trained experimenter. Surveys, olfactory assessments and health assessments were conducted in a separate session which was led by a nurse. Each session lasted for up to 2 h^19^.

### Participants

**Figure 1:**
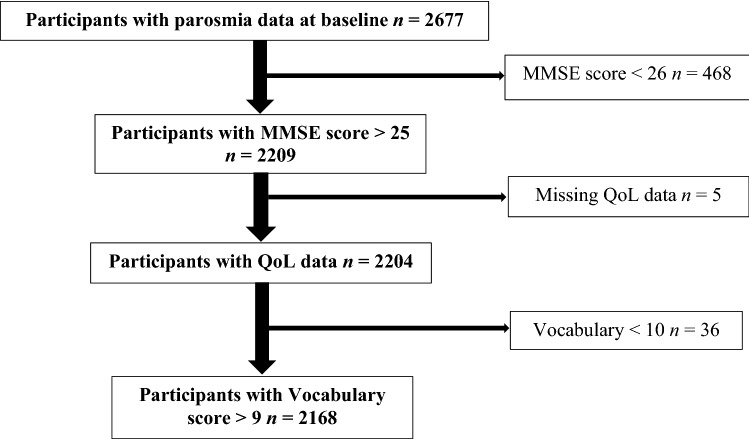
Exclusion flowchart. MMSE, Mini-Mental State Examination. QoL, Quality of Life. Vocabulary, Vocabulary test.

Participants in the Betula study were recruited from the municipality of Umeå, Sweden. They were randomly selected from the population registry, stratified by age and gender. The participants were recruited to enable a narrow-age cohort design with 5-year intervals (i.e. at time of testing, participants were of the ages 35, 40, 45 … 90 years) and the number of females and males selected for inclusion was proportional to the general population's female-male ratio in each age-group. The total data set for the current study (third, fourth and fifth wave) contained 4712 participants. For this study, individuals who did not participate in the third testing wave, or who did not provide data on the question regarding parosmia, were excluded (*n* = 2035),  leaving 2677 participants. In addition, participants scoring under the cut-off value of 26 points (*n* = 468) on the Mini-Mental State Examination^22^ with missing data on the QoL-questionnaire (n = 5) or with vocabulary assessment results three standard deviations lower than the group mean (*n* = 36) were excluded (see Fig. 1). Thus, the final study sample consisted of 2168 individuals (between 35 and 90 years of age, 53.2% female). Note that in our analyses, there are a few additional missing data points that change the degrees of freedom slightly across analyses. The study was conducted in accordance with the Declaration of Helsinki and approved by the Ethics Committee of the Faculty of Medicine and Odontology, Umeå University. All participants gave their written informed consent.

### Assessment of parosmia

In the Betula study, parosmia was assessed through self-reports with the question “Does it happen that you smell something, for example a rose or an orange, that should have a smell that you recognize, but instead you feel a bad, tainted or burned smell?”. The question was answered with either “cannot”, “no” or “yes”. Participants were grouped as parosmics (yes) and non-parosmics (no).

### Correlates of parosmia

#### Demographic variables

Demographic variables included sex, age and years of education, which was reported in 0.5-year intervals. Mean differences in age and educational background between the parosmic and non-parosmic population were examined.

#### Olfactory functions

In the Betula study, self-rated assessments of olfactory abilities were acquired through a questionnaire consisting of four questions concerning the respondent’s ability to feel weak odors, parosmic and phantosmic experiences, and presence of diseases in the nose (at the time of the questionnaire). Ability to feel weak odors was examined through the question “How is your ability to feel weak odors?”. The question was answered with either “cannot”, “worse than normal”, “normal” or “better”. Participants were grouped as worse than normal (cannot, worse than normal) or normal (normal, better). Phantosmia was assessed with the question “Does it happen that you feel a bad, tainted or burned smell when there is nothing that could have caused it?”, and the presence of a nose disease with the question” At present, do you have some type of disease in the nose, for example problems to breathe through the nose, infection in sinus, nasal polyps, or allergic symptoms in the nose?”. The questions were answered with either “cannot”, “no” or “yes”. If there was a disease in the nose prevalent, participants were able to state what sort of disease it was. The Betula study includes a version of the Scandinavian Odor Identification Test (SOIT)^23^. This version comprises 13 olfactory stimuli that are familiar to the Scandinavian population: pine-needle, juniper berry, violet, anise, clove, vanilla, almond (bitter), orange, cinnamon, lemon, lilac, tar, and apple. The intensity of the odors is fairly strong and a wide range of odor qualities are represented, such as floral, citrus, non-citrus fruity, sweet, woody, and spicy. Participants were presented a written list of four response alternatives for each stimulus, where one verbal label correctly represented the odor^24^. Each participant was randomly presented with one out of 10 different orders of the 13 olfactory stimuli. The odors and their corresponding response alternatives were identical for all participants. There was a 30 s inter-stimulus interval between odor items, to prevent effects of adaptation. SOIT has a low- to moderate correlation with several cognitive tests^24^.

#### Cognitive functions

To screen for individuals with cognitive deficits which might affect the overall reliability in test- and questionnaire results, a vocabulary test and the MMSE were used. The vocabulary test used in the Betula study was a 30-item multiple-choice test consisting of one target word and 5 alternatives, where the instructions were to identify the synonym to the target word out of the 5 alternatives^25^. The test was self-paced with a time limit of 7 min. Hence the vocabulary test resembled the SOIT in that both tests used a multiple-choice format of matching a stimulus to one of several verbal response alternatives. In order to control for general cognitive deficits which might indicate dementia, the Mini-Mental State Examination was used. The MMSE tests cognitive ability through examining the individual’s orientation, perception, attention and calculation, recall, and language abilities^22^. Both tests were included as possible predictors of self-reported parosmia.

#### Quality of Life

The Betula study included a questionnaire consisting of 21 questions assessing subjective health status. Out of those, 5 questions were selected for the present study as a relevant measure of quality of life.

1. Do you feel well?
2. Are you troubled by loss of appetite?
3. Are you troubled by often feeling dispirited?
4. Are you troubled by often feeling lonely?
5. Are you troubled by anxiety, worries?

This set of questions were chosen because of their similarities to questions and questionnaires regarding mental health and quality of life used in other studies of olfactory dysfunction^3,4,17,18^. All questions could be answered with either “no” or “yes”. Question 1 was reverse-coded. A higher value on the combined mean-score for the QoL-questions (QoL-sum) indicates a worse quality of life (intervals 0–5).

### Statistical analysis

Hierarchical logistic regression analyses were used to examine the relationship between parosmia and the included predictors at baseline. The first block in the regression model (block 1) consisted of sex, age and educational background. Latter blocks consisted of olfactory functions (block 2) and cognitive ability (block 3). Nagelkerke’s pseudo-R2 is reported for every step, as a measure of model fit. Omnibus chi-square test of model coefficients was used to test if the inclusion of additional blocks resulted in significantly improved model fit. Odds ratios (OR) including 95% confidence intervals (CI) and p-values for each contrast are reported. A *t*-test was used to compare the mean scores of the sum of the 5 QoL-questions between the parosmic and non-parosmic groups. Additional hierarchical logistic regression analyses were conducted with the purpose of investigating if the variables for the parosmic group at baseline had a relationship with the outcome of parosmia at the 5-year follow-up. This was to determine whether any variables could predict the persistence of parosmia at the 5-year follow-up. All statistical analyses were performed with the SPSS statistical software package (version 27.0 for Mac).

## Results

The overall prevalence of parosmia was 4.8% (*n* = 104 of 2168). Descriptive data for demographic and behavioral variables is presented for the non-parosmic and parosmic groups at baseline in Table 1. In order to identify which baseline-variables that were associated with parosmia at baseline, a block-wise hierarchical logistic regression analysis was performed. The hierarchical logistic regression results are presented in Table 2. This analysis was based on a sample of 2115 individuals, as 53 of the 2168 included participants (2.4%) had missing data for at least one of the variables (most often years of education). They were excluded from this analysis but they were included in the study at large (see Fig. 1 for our screening procedure). The demographic variables in block 1 were not significantly associated with parosmia (*p* = 0.06). When olfactory variables were added in block 2, the model fit significantly improved (Nagelkerke’s pseudo-R2 = 0.151; *p* ≤ 0.001). After including also cognitive variables, the model was not improved further (Nagelkerke’s pseudo-R^2^ = 0.155; *p* ≤ 0.001). In the final regression model, only three significant individual correlates were identified, all from block 2 (olfaction). Prevalent phantosmia was strongly associated with parosmia (*p* ≤ 0.01), as 42.3% of parosmics, but only 6.9% of non-parosmics reported phantosmia. Disease in the nose was more often present in parosmics (24.0%) than in non-parosmics (16.2%; *p* ≤ 0.05). Self-reported inability to perceive weak odors was not related to parosmia. We investigated manually whether people might have reported their parosmia as a current disease of the nose, which would trivially explain the association between these two variables, by reviewing a free text variable where the disease of the nose was specified (notes by test administrator during the interview). A majority of those who reported having a disease of the nose reported a common cold, nasal congestion or allergies, but no person reported parosmia as the present nasal disease. This excludes the possibility that the statistical association between parosmia and disease of the nose were due to incorrect reporting. Surprisingly, higher SOIT-scores also showed an association with parosmia (*p* ≤ 0.05). None of the demographic or cognitive variables contributed significantly to parosmia. Parosmia was thus associated with phantosmia, disease of the nose, and, surprisingly, a marginally higher odor identification performance level (see Table 1).

**Table 1:** Demographic and behavioral characteristics at baseline.

Characteristics	Parosmia	
	No (n = 2064)	Yes (n = 104)
**1. Demographics**		
Sex (female/male)	1101/963	52/52
Age (M ± SD)	61.19 ± 13.06	62.07 ± 13.47
Education (M ± SD)	11.03 ± 4.21	9.99 ± 3.69
**2. Olfaction**		
Ability to feel weak odors (normal/worse)	1640/424	78/26
Phantosmia (no/yes)	1921/143	61/43
Disease in nose (no/yes)	1728/335	79/25
SOIT (M ± SD)	7.22 ± 2.15	7.49 ± 2.27
**3. Cognition**		
Vocabulary (M ± SD)	23.14 ± 4.11	22.15 ± 4.38
MMSE (M ± SD)	28.19 ± 1.19	28.06 ± 1.33

*M* mean, *SD* standard deviation, *MMSE* mini-mental state examination, *SOIT* Scandinavian odor-identification test.

**Table 2:** Block wise hierarchical logistic regression analysis for correlates of parosmia.

Characteristics	Nagelkerke’s pseudo-R^2^	Model parameters		Model parameter, all factors included	
		OR	95% CI	OR	95% CI
**1. Demographics**	0.011				
Sex		1.11	0.75–1.66	1.39	0.90–2.13
Age		0.99	0.97–1.01	0.99	0.97–1.01
Education		0.92*	0.87–0.98	0.94	0.87–1.01
**2. Olfaction**	0.151ª**				
Ability to feel weak odors		0.86	0.52–1.42	0.86	0.52–1.43
Phantosmia		9.95**	6.43–15.42	9.97**	6.42–15.50
Disease in nose		1.71*	1.03–2.83	1.75*	1.06–2.91
SOIT		1.12*	1.01–1.25	1.13*	1.02–1.26
**3. Cognition**	0.155				
Language		0.97	0.92–1.03	0.97	0.92–1.03
MMSE		0.91	0.76–1.10	0.91	0.76–1.10

*MMSE* mini-mental state examination, *SOIT*, Scandinavian odor-identification test, *OR* odds ratio, *95% CI* 95% confidence interval.

*p < 0.05, **p < 0.01.

ªSignificance was based on the omnibus chi-square test of model coefficients.

A *t*-test was used to investigate potential differences between the parosmic and non-parosmic populations’ sum of the QoL scores. There was no evidence of unequal variances between the groups (Levene’s *p* = 0.53), and the *t*-test showed no significant differences of the sum of the QoL-questions, *t*(2166) = − 0.94, *p* = 0.35, between the parosmic (*M* = 0.70, *SD* = 0.99) and non-parosmic groups (*M* = 0.61, *SD* = 0.94), with a very small estimated effect size (Cohen’s *d* = 0.09). Exploratory follow-up t-tests indicated that there were no significant group differences for any of the 5 individual QoL-questions (all *p*s ≥ 0.34). There was thus no meaningful difference between the parosmic and non-parosmic groups in self-rated QoL.

Next, we assessed how long the parosmia symptoms lasted, using data from follow-up test waves. A total of 946 individuals (constituting 43.6% out of the original 2168 participants) participated in the 5-year follow-up and of those, 47 (5.0%) of the participants had parosmia at baseline. The smaller sample at follow-up is mostly due to the fact that the Betula study size varies across testing waves and not all participants are asked to return. Dropout is also present in all longitudinal studies, but Betula has a very high return rate overall, and the most common reason for dropping out is that participants are deceased, the risk of which is higher in older age groups^24^. In Table 3 the sample sizes from baseline, the 5-year follow-up, and the ten-year follow-up are presented. At the 5-year follow-up, 45 (4.8%) individuals had self-rated parosmia, of which eight (17.8%) had parosmia at baseline and 37 (82.2%) had developed parosmia since baseline. Of the 47 individuals with parosmia at baseline who returned for the 5-year follow-up, 39 (83.0%) had recovered and 8 (17.0%) had not recovered during the 5-year span. There were also many new cases of parosmia. Of 899 participants at the 5-year follow-up who had reported not having parosmia at baseline, 37 (4.1%) had now developed parosmia. We investigated demographic, olfactory and cognitive variables at baseline that might predict the persistence of parosmia (self-rated parosmia at baseline and the 5-year follow-up) through a block-wise hierarchical logistic regression analysis. Results showed that phantosmia was the only significant predictor (OR = 16.13, *p* = 0.025, 95% CI 1.42–183.45). Descriptive data also indicates an association between baseline phantosmia and sustained parosmia; of the 8 participants who went on to have persistent parosmia, 6 were phantosmic at baseline (75%), whereas of the 39 parosmics that recovered, only 14 (36%) were phantosmic at baseline. Having parosmia and phantosmia simultaneously increases the risk of having parosmia 5 years later.

**Table 3:** Number of participants with and without parosmia at each test-wave, and their parosmia status at the following test-wave.

	Parosmia	Will retain	Will recover	No follow-up	No parosmia	Will aquire	No follow-up
Baseline (n = 2168)	104	8	39	57	2064	37	1165
+ 5 years (n = 946)	45	4	29	12	901	16	220
+ 10 years (n = 714)	20	*–*	*–*	(all)	693	*–*	(all)

*Will retain* parosmics who will retain their parosmia at the next testing occasion, *Will recover* parosmics who will recover at the next testing occasion, *Will aquire* non-parosmics who will aquire parosmia by the next testing occasion, *No follow-up* participants who were not involved in future testing. Note that at 10-year testing, further follow-up data is not available.

There was also data from a ten-year follow-up, where 5 (12.8%) out of 39 individuals who had parosmia at baseline still had parosmia at the 10-year follow-up. Out of the 8 individuals who had had parosmia at baseline and at the 5-year follow-up, only 6 participated in the 10-year follow-up. Four out of these 6 had parosmia at the 10-year follow-up (10.3% out of the 39 baseline parosmics). This means that 4 (10.3%) out of the 39 individuals who had parosmia at baseline, and who were followed for 10 years prospectively, had reported parosmia at all three assessments over 10 years. This might indicate that if parosmia does not pass within 5 years, it is likely to still be prevalent after 10 years. This interpretation was supported by a chi-square statistic with Yates correction which gave the result that the parosmia recovery was not significant in the 5–10 year follow-up interval, *X*^*2*^ (1, 39) = 0.59, *p* = 0.44. Interestingly, one individual reported no parosmia at the 5-year follow-up, but had been affected by parosmia again at the ten-year follow-up. Table 3 also shows that the overall prevalence of parosmia was stable at baseline and 5-year follow-up but was lower at the 10-year follow-up, which could be due to either random fluctuations or attrition effects.

## Discussion

We investigated experiences of parosmia with the aim of understanding how they affect the general population; this may inform recovery prognoses for those similarly affected during the COVID-19 pandemic. Our study had 5 key features. First, the prevalence of parosmia was investigated in a population-based sample. Second, the relationship between parosmia and demographic variables, and third, the relationship between parosmia and other olfactory variables were investigated. Fourth, the parosmics’ self-rated quality of life was compared to that of the non-parosmic sample. Lastly, the persistence of parosmia over 5 (and ten) years was investigated.

The results showed that 4.8% of the population between 35 and 90 years of age had parosmia. The prevalence of phantosmia and a current nose disease, as well as somewhat higher scores on the Scandinavian odor identification test, was associated with parosmia. Parosmia was not associated with self-rated odor perception. Surprisingly, the parosmic population did not exhibit lower scores on QoL, and did not report a reduced appetite. Our prevalence estimates correspond to those obtained in a previous population-study in Sweden where 4.0% of the population 20 years and older reported having parosmia^2^. The current study excluded individuals with low scores on MMSE and the language-test to control for inaccurate self-ratings of parosmia due to misunderstanding of the question. Despite this, one can argue that the current study may be liable to over-diagnosing parosmia due to the fact that some individuals might have interpreted the question wrongly. In the most similar previous study, parosmia was evaluated through an interview with a clinical professional, and the previous result was similar to the present estimate^2^. Other researchers have concluded, in contrast, that the prevalence of parosmia might be below 2%^8^. Future work should provide multiple questions regarding parosmia to get a more reliable assessment. However, as the results were congruent with prior findings from similar work, they strengthen the view that parosmia might have been a rather common symptom in the general public, even before COVID-19. We do not have detailed data on the strength, persistence or specific triggers associated with the parosmic experiences, and this should be regarded as a limitation. Individuals who are only occasionally or mildly affected by parosmic sensations are likely to be included in our sample, and thus our sample should be regarded as distinct from clinically recognized parosmia patients.

Little is known about the longevity of parosmia. This is the first study, to our knowledge, to present prospective, population-based data on the persistence of parosmia over 5 and 10 years. Our results show that a sixth (17.0%) of individuals who reported parosmia at baseline had parosmia 5 years later, and a tenth (10.3%) still reported parosmia at the ten-year follow-up. Although our long-term estimates of parosmia are affected by a shrinking sample size, the results suggest that having parosmia for 5 years means that it will likely persist over 10 years as well. Concurrent phantosmia was a significant predictor of the risk of the parosmic condition becoming chronic. We propose that the parosmic population in general may not experience a significantly reduced quality of life, compared to the non-parosmic population. Previous research has mainly focused on clinical populations (i.e. patients at smell and taste clinics), where results indicate that having an OD (including parosmia) is connected to also having symptoms of depression, or a lowered quality of life^3,4,9,13^. One explanation to why the results of this study do not fit with those of previous studies is that the current study is based on a population-based sample instead of clinical samples. Those who seek medical attention for parosmia is likely to do so because their quality of life is impaired. In contrast, our results may arguably be more representative of the experiences of olfactory distortions in the population as a whole, which probably includes a large proportion of mild cases. One implication of our current findings is that research on the different manifestations of parosmia may be investigated along with the use of coping strategies, in order to better understand how some individuals may successfully adapt in the long term^26,27^.

The general consensus in the research field seems to be that parosmia in clinical samples is heavily associated with reduced olfactory functioning^4–6,9,13^. The results from this study suggests instead that parosmia might be experienced in the general population independently of quantitative ODs. The most consistent association observed in this study was that between parosmia and phantosmia, corroborating previous work^12^. This study has limitations which need to be considered when interpreting the findings presented. First, as mentioned earlier, the fact that parosmia was evaluated through one questionnaire question is a limitation of the study, since it relies heavily on the participants' language comprehension and interpretation of their own olfactory function. Second, the baseline data collection for this study was conducted between 1998 and 2000 and extrapolations to the current COVID-pandemic context should be tempered. Third, the prevalence of parosmia report decline nominally from 4.8% at baseline and 5-year follow-up to 2.9% at the 10-year follow-up, which might make the results of the last follow-up less robust, especially when considering the smaller sample overall. It is possible that parosmics might, for reasons unknown to us, be more inclined to drop out of testing, but this would likely have led to an observed prevalence reduction already at the 5-year follow-up, which was not observed. Thus, we attribute the late decline of parosmia prevalence to random fluctuations in our decreasing sample. Fourth, this quantitative study neither addresses the individual parosmic experiences, nor does it explain the causes of parosmia. Although our results indicate that parosmics in general do not suffer more due to their disorder then the non-parosmic population, this does not mean that all individual cases follow the same pattern. There are certainly cases where parosmia leads to long-lasting suffering, but our results suggest that in the general population, the average parosmia case might be less severe. Future large-scale surveys on parosmia might include questions about presumed causes and durations of symptoms.

Further research is needed regarding the range of intensity and impact of experiences of parosmia, considering that our results show it may last for over 10 years, and that there are reports of parosmia persisting up to 22 years^9^. Since parosmia varies in duration, it raises the question whether there is a difference between the severity of symptoms and experienced suffering between those with short-term and long-term parosmia. The possible interactions between parosmia and phantosmia also need further investigation. Importantly, further research should consider parosmia as a spectrum, and strive to develop effective instruments that can better evaluate the varying severity of parosmia^28^.
